# Complete Genome Sequence of Buttiauxella agrestis DSM 9389

**DOI:** 10.1128/MRA.00301-21

**Published:** 2021-05-13

**Authors:** Nao Nakamichi, Ryota Moriuchi, Hideo Dohra, Hiroyuki Futamata, Yosuke Tashiro

**Affiliations:** a Department of Engineering, Graduate School of Integrated Science and Technology, Shizuoka University, Hamamatsu, Japan; b Research Institute of Green Science and Technology, Shizuoka University, Suruga-ku, Shizuoka, Japan; c Department of Science, Graduate School of Integrated Science and Technology, Shizuoka University, Shizuoka, Japan; d Graduate School of Science and Technology, Shizuoka University, Hamamatsu, Japan; e JST PRESTO, Kawaguchi, Saitama, Japan; University of Maryland School of Medicine

## Abstract

We report here the complete genome sequence of Buttiauxella agrestis DSM 9389, which harbors eight 16S rRNA genes classified into three types. The genome sequence of this strain showed a high average nucleotide identity (97.3%) with that of the highly membrane vesicle-producing strain *B. agrestis* ATCC 33320^T^.

## Abstract (precis)

We report here the complete genome sequence of Buttiauxella agrestis DSM 9389, which harbors eight 16S rRNA genes classified into three types. The genome sequence of this strain showed a higher average nucleotide identity value (97.3%) than that of the highly membrane vesicle-producing strain *B. agrestis* ATCC 33320^T^.

## ANNOUNCEMENT

Buttiauxella agrestis DSM 9389 (S3/6-333), which was isolated from a slug sampled in Braunschweig, Germany (1), is a member of the family *Enterobacteriaceae*. While some *Buttiauxella* spp. have unique characteristics in membrane vesicle formation (2, 3), genomic information of the species is limited to several reports (4, 5). In this report, we announce the DSM 9389 complete genome sequence and confirm the species with average nucleotide identity (ANI).

**FIG 1:**
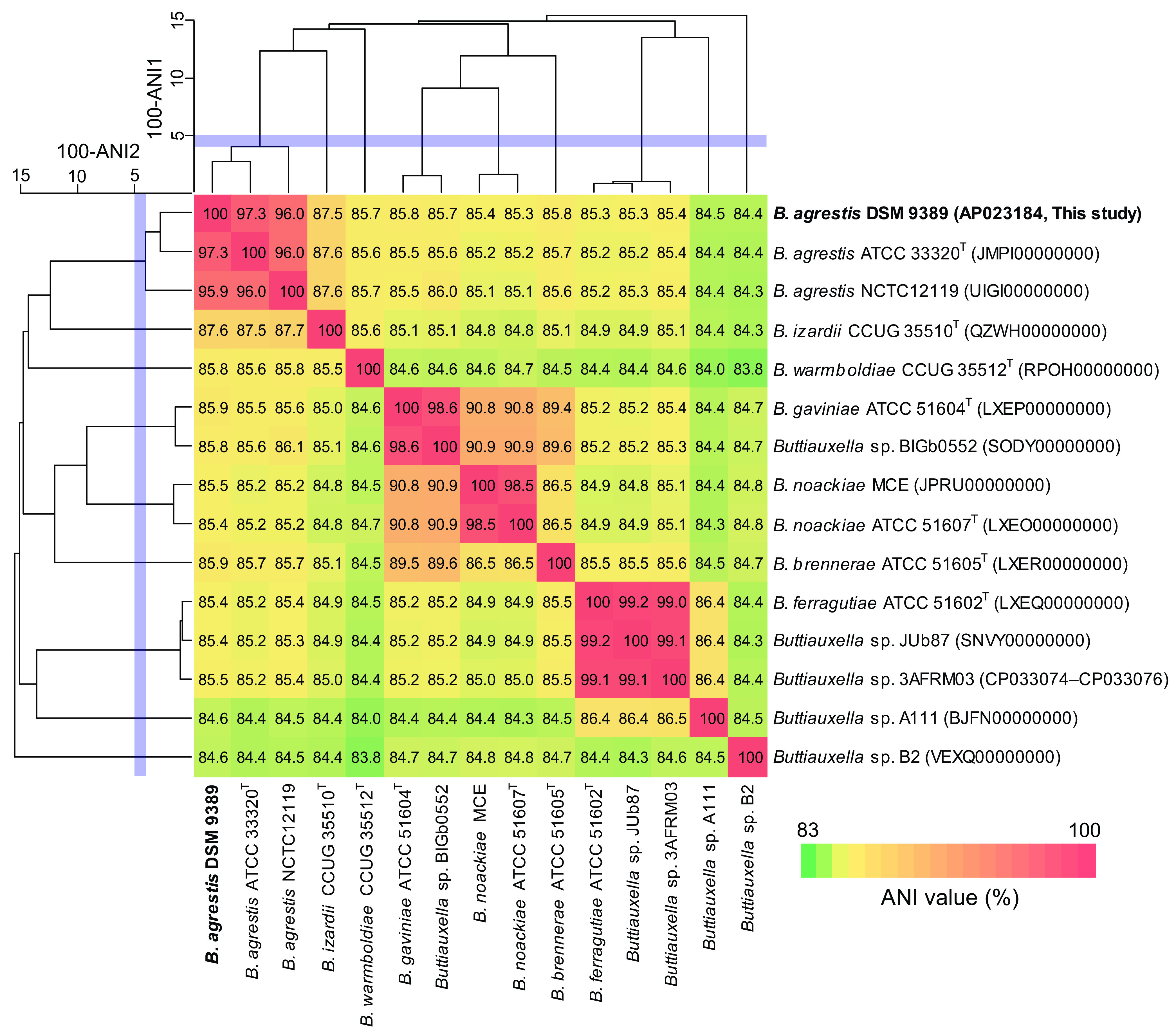
Average nucleotide identity (ANI) matrix of the genome sequences for *B. agrestis* DSM 9389 and related species. ANI values are visualized by the heatmap, and the relationship of the strains for ANI values are shown by the dendrogram. Blue bands represent the species threshold for ANI values of 95% to 96% (15, 16). GenBank accession numbers for the genome sequences used in this analysis are shown in parentheses.

*B. agrestis* DSM 9389 was grown in LB medium at 30°C for 16 h, and its genomic DNA was extracted using a Wizard genomic DNA purification kit (Promega). The complete genome sequence of *B. agrestis* DSM 9389 was determined by the combination of PacBio long reads and Illumina short reads. The genomic DNA was sheared using g-TUBE (Covaris) and size selected using the BluePippin system with a High Pass Plus cassette (Sage Science). A PacBio 20-kb library was prepared using the SMRTbell template prep kit and sequenced on the PacBio RS II instrument (Pacific Biosciences) at Macrogen, Inc. (Seoul, South Korea). An Illumina library was constructed using a TruSeq Nano DNA library prep kit and sequenced on the Illumina MiSeq platform (301-bp paired-end sequencing). Information on the PacBio and Illumina reads used in this study is summarized in Table 1. PacBio subreads were filtered (length, ≥6,000 bp; read quality, ≥0.85) using BamTools v. 2.4.1 (6), and the long and high-quality reads were assembled using Canu v. 1.8 (7). The resulting single contig was polished using Arrow v. 2.2.2 (https://github.com/PacificBiosciences/GenomicConsensus) and then circularized and rotated to start with the *dnaA* gene using Circlator v. 1.1.1 (8). Illumina reads were cleaned up by trimming adapter sequences and low-quality ends (quality score, ≥15; read length, ≥150 bp) using Trimmomatic v. 0.38 (9). The high-quality reads were aligned to the polished contig using BWA-MEM v. 0.7.15 (10), and assembly errors were corrected using Pilon v. 1.23 (11). Default parameters were used except where otherwise noted. The complete genome sequence of *B. agrestis* DSM 9389 consisted of a circular chromosome of 4,566,254 bp with a G+C content of 50.7%. The genome was annotated using DFAST v.1.2.3 (12). The genome contains 4,110 protein-coding sequences, 25 rRNA genes, and 83 tRNA genes. Of the eight 16S rRNA genes of strain DSM 9389, five were identical to those of Buttiauxella noackiae NSW 11 (GenBank accession number NR_036919.1), and no gene was identical to that of *B. agrestis* ATCC 33320^T^ (NR_041968.1). To confirm the species definition, ANI analysis (13) was performed using a ruby script (ani.rb) from the enveomics collection (14). The genome sequence of *B. agrestis* DSM 9389 showed a high ANI (97.3%) with that of strain *B. agrestis* ATCC 33320^T^ (Fig. 1), resulting in our conclusion that *B. agrestis* DSM 9389 was confirmed to be *B. agrestis*.

**TABLE 1:** Summary of reads generated by the MiSeq and PacBio platforms

Parameter	Data for:	
	Illumina MiSeq	PacBio RS II
Raw reads^*a*^		
No. of reads	2,020,870	103,022
Total bases (bp)	605,620,107	1,143,739,721
*N*_50_ (bp)	16,358	
Filtered reads		
No. of reads	1,743,456	47,499
Total bases (bp)	461,379,893	723,912,023
*N*_50_ (bp)	17,225	
Mean coverage (×)^*b*^	101.0	158.5
Accession no.	DRR226681	DRR226682

a Raw reads from PacBio RS II indicate subreads with a read quality of ≥0.75.

b Total bases of filtered reads (base pairs)/genome size of DSM 9389 (base pairs).

### Data availability.

The sequence reads have been deposited in the DDBJ Sequence Read Archive (DRA)/SRA under the accession numbers DRR226681 (Illumina MiSeq) and DRR226682 (PacBio RS II). The complete genome sequence of *B. agrestis* DSM 9389 has been deposited in DDBJ/ENA/GenBank under the accession number AP023184.
